# Editorial: Verification of Animal Pain Models by Reverse Translation

**DOI:** 10.3389/fphar.2021.778880

**Published:** 2021-10-08

**Authors:** Robert M. Caudle, Maree Therese Smith, E. Alfonso Romero-Sandoval

**Affiliations:** ^1^ Department of Oral and Maxillofacial Surgery, University of Florida, Gainesville, FL, United States; ^2^ School of Biomedical Sciences, The University of Queensland, Brisbane, QLD, Australia; ^3^ Wake Forest School of Medicine, Winston-Salem, NC, United States

**Keywords:** nociception, animal models, analgesic, behavioral testing, bedside to bench

**FIGURE 1:**
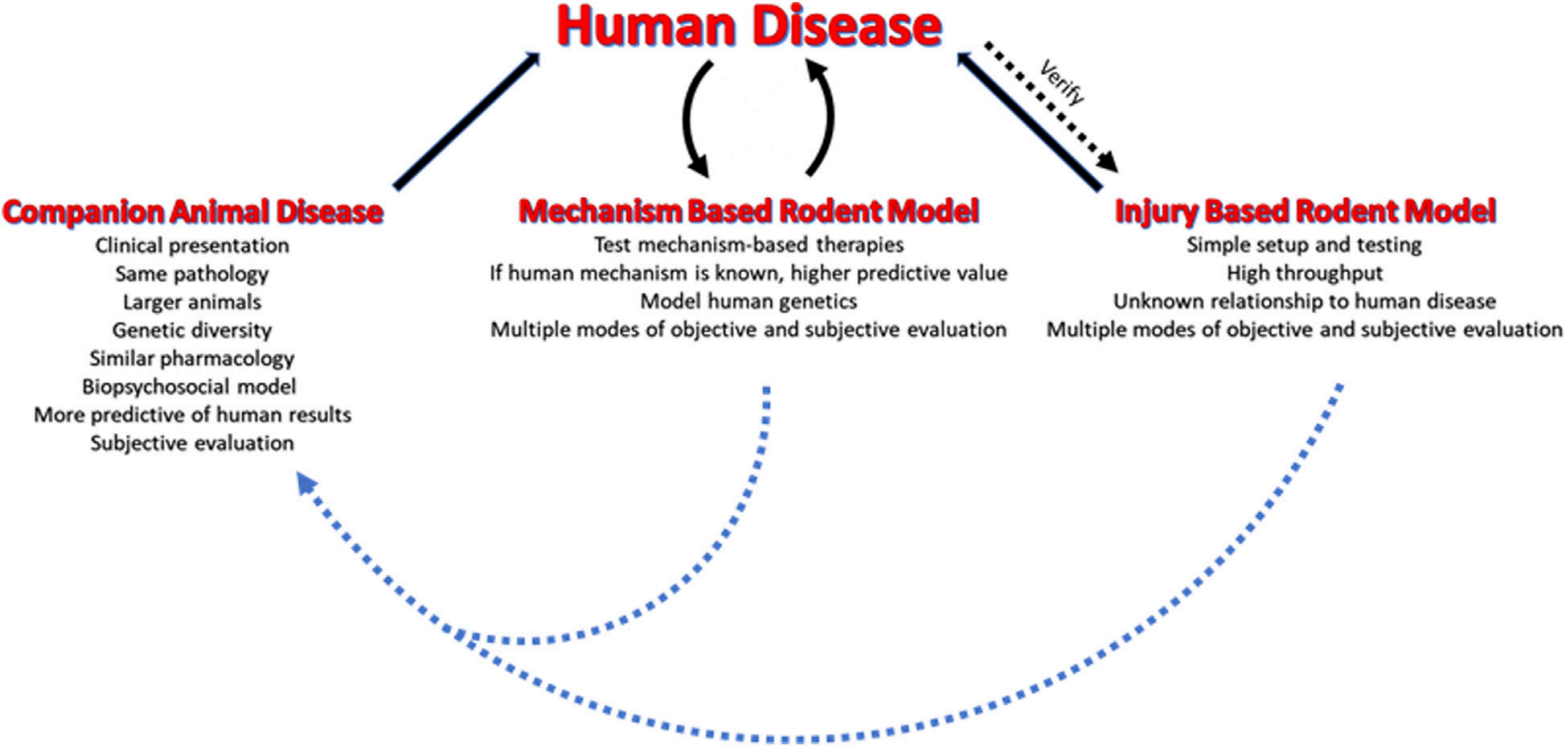
Modeling human pain disorders in animals. Rodent injury models have been the standard for pain research and therapy evaluation. These models need to be compared to the human disease they model to ensure that they are predictive. The mechanism based rodent models are theoretically superior to the injury models due to their focus on specific mechanisms for producing pain. These models are developed based on known or suspected causes of the human disease. The companion animal models are most closely related to human disease and can bridge the gap between rodent studies and humans. If possible, it is recommended that companion animal clinical trials be conducted after the rodent studies and prior to initiating human trials.

The validity of preclinical models in evaluating and developing potential analgesics has been an area of discussion for over a decade. The poor success of these models in predicting clinical efficacy (Woolf, 2010; Barrett, 2015; Burma et al., 2017) leads to the question as to whether the fault lies in the models not invoking the same pathology as the diseases being modeled, or if the failure is due to the differences in the physiology, anatomy, and pharmacology of the model species versus humans. An additional factor is that the design of proof-of-concept clinical trials of novel analgesic agents may have been sub-optimal (Hijma and Froeneveld, 2021). Ideally, the animal models being used for analgesic discovery would have the same pathophysiology as the human disease and would predict with 100 percent accuracy the efficacy of an agent in human trials. This ideal, however, is not realistic and compromises are necessary. However, repeated verification and optimization of the models can inform investigators of the limitations of the models, and by utilizing multiple models a realistic evaluation of the therapeutic potential of an agent can be realized. This research topic is devoted to outlining methods to increase confidence in the translatability of the data obtained in animal models of human pain conditions. An important aspect of this process is to use known information about human disease to improve the animal models, create new models, and to eliminate non-productive models. The papers in this research topic can be divided into three general categories of discussion (see Figure 1). The first category evaluates rodent models by reverse translation. Known therapies or known characteristics of the disease in humans are examined in established models to determine if there is a correlation between humans and rodents. The review by Fisher et al. evaluates neuropathic pain models and argues that matching quantifiable endpoints between the model and humans is critical to improving the translatability of the data obtained from the model. They further suggest, as do several of the authors in the topic, that pain suppressed behaviors may be superior to pain enhanced behaviors when determining therapeutic potential of an agent. The original research by Negus et al. addresses the affective/motivational aspect of pain when using a rodent model. In humans the distress induced by pain is often the most critically relevant feature of pain that determines the duration of disability or the quality of life for a patient. The authors present data indicating that rodents have a weak affective/motivational response to injury, suggesting that rodents may only model the sensory/discriminative aspect of pain; thus, making them less relevant as human models. This conclusion is countered by Cho et al. who suggest that a complete evaluation of the biopsychosocial model of pain in rodents is possible and can lead to a better understanding of pain and pain control in humans. They present methods of evaluating rodent behavior that provide insight into the psychological and social aspects of pain. The review by Pineda-Farias et al. evaluates models of cancer pain and provides hope that greater knowledge of how cancer and cancer therapy produce pain in humans will inform modeling in animals. The interesting review by Shen et al. examines complex multi tissue injuries associated with damaged bone. The authors argue that understanding traumatic injury in humans is necessary for developing animal models that are informative for therapy evaluation. Finally, the original research by Caudle et al. reexamines a model of migraine using an operant orofacial pain assay in rats to demonstrate that standard migraine rescue agents do not work in this model. Their findings indicate that the model may be inadequate for evaluating therapeutics for migraine headache. The second category of papers utilizes known facts about human disease to create novel more representative models of the disease. Castañeda-Corral et al. examine type 2 diabetes induced painful peripheral neuropathy in rodents by feeding a “cafeteria style” diet to rodents. The diet mimics a western diet, inducing weight gain, increasing blood sugar, and sensitizing the rodents to streptozotocin induced diabetes. The resulting diabetes produces a slow onset neuropathy that has many characteristics of human type 2 diabetes painful peripheral neuropathy. The work by Levine et al. creates a model of migraine headache using data from human studies suggesting that there is a dysregulation of the endocannabinoid system in migraineurs. Blocking diacylglycerol lipase, a key synthetic enzyme for generating 2-arachidonoylglycerol, produced facial mechanical allodynia and photophobia. Critically, there was no paw sensitivity produced by diacylglycerol lipase blockade indicating that the model was specific for trigeminal regions. The authors further demonstrated that the allodynia could be reversed with clinically used rescue agents. In Cho et al. the authors suggest that engineering models that genetically match humans with the disease and identifying ways to evaluate pain memory and affect are critical to improving rodent models. The final category in this topic describes using companion animals that have naturally contracted disease. Iadarola et al. provide an overview of their use of canine osteosarcoma and osteoarthritis patients to evaluate novel pain therapeutics following extensive testing in multiple rodent models. Similarly, Cho et al. consider companion animals as an intermediate step between rodents and humans. Both sets of authors indicate that these veterinary clinical trials provide strong verification of efficacy, dosing information, and toxicological information that significantly reduces the probability of failure in a human trial. Overall, the authors in this topic remain optimistic that the problems with animal models can be resolved with extensive feedback from human studies that inform the refinement of old models and the design of new models. It is also becoming clear that multiple rodent models and testing methods may be needed to capture the full complexity of human disease when assessing new agents, and routinely incorporating companion animal clinical trials, when possible, could further enhance the success rate of human trials.
